# Metabolomic signature of amino acids in plasma of patients with non-segmental Vitiligo

**DOI:** 10.1007/s11306-021-01843-x

**Published:** 2021-09-25

**Authors:** Rezvan Marzabani, Hassan Rezadoost, Peyman Choopanian, Sima Kolahdooz, Nikoo Mozafari, Mehdi Mirzaie, Mehrdad Karimi, Anni I. Nieminen, Mohieddin Jafari

**Affiliations:** 1 Department of Phytochemistry, Medicinal Plants and Drugs Research Institute, Shahid Beheshti University, Tehran, Iran; 2 Department of Applied Mathematics, Faculty of Mathematical Sciences, Tarbiat Modares University, Tehran, Iran; 3 School of Traditional Medicine, Tehran University of Medical Sciences, Tehran, Iran; 4 Skin Research Center, Shahid Beheshti University of Medical Sciences, Tehran, Iran; 5 Research Program in Systems Oncology, Faculty of Medicine, University of Helsinki, 00290 Helsinki, Finland; 6 Metabolomics Unit, Institute for Molecular Medicine Finland (FIMM), HiLIFE, University of Helsinki, 00290 Helsinki, Finland

**Keywords:** Vitiligo, Plasma, Metabolomics, Amino acids, Liquid chromatography

## Abstract

**Introduction:**

Vitiligo pathogenesis is complicated, and several possibilities were suggested. However, it is well-known that the metabolism of pigments plays a significant role in the pathogenicity of the disease.

**Objectives:**

We explored the role of amino acids in vitiligo using targeted metabolomics.

**Methods:**

The amino acid profile was studied in plasma using liquid chromatography. First, 22 amino acids were derivatized and precisely determined. Next, the concentrations of the amino acids and the molar ratios were calculated in 31 patients and 34 healthy individuals.

**Results:**

The differential concentrations of amino acids were analyzed and eight amino acids, i.e., cysteine, arginine, lysine, ornithine, proline, glutamic acid, histidine, and glycine were observed differentially. The ratios of cysteine, glutamic acid, and proline increased significantly in Vitiligo patients, whereas arginine, lysine, ornithine, glycine, and histidine decreased significantly compared to healthy individuals. Considering the percentage of skin area, we also showed that glutamic acid significantly has a higher amount in patients with less than 25% involvement compared to others. Finally, cysteine and lysine are considered promising candidates for diagnosing and developing the disorder with high accuracy (0.96).

**Conclusion:**

The findings are consistent with the previously illustrated mechanism of Vitiligo, such as production deficiency in melanin and an increase in immune activity and oxidative stress. Furthermore, new evidence was provided by using amino acids profile toward the pathogenicity of the disorder.

**Supplementary Information:**

The online version contains supplementary material available at 10.1007/s11306-021-01843-x

## Introduction

Vitiligo is a common chronic skin disorder in which pigment-producing cells or melanocytes are unregulated, resulting in varying patterns and degrees of skin depigmentation (Armstrong, 2011; Sahoo et al., 2017). It is common, affecting 0.5–2% of the world’s population without preference for race or sex (Rodrigues, M., K. Ezzedine, I. Hamzavi, A. G. Pandya, J. E. Harris & V. W. Group, 2017). Diagnostically, patterns of lesions in vitiligo predict progression and treatment responses (Ezzedine et al., 2012). Vitiligo manifests as multiple spots appearing on the skin in a symmetric pattern on both sides of the body. It may impact only mucosal parts of the body (mucosal vitiligo), lips or other parts of the face plus the hands and/or feet (acrofacial vitiligo), lips and fingertips (lip-tip vitiligo), only affecting a small area of the body (focal vitiligo), involve large parts of the body all over (generalized vitiligo), and most of the body (universal vitiligo). In addition to different anatomical locations on the body, there are also different appearances that the spots themselves can have. Four typical appearances include: inflammatory, trichrome, or confetti vitiligo, and Koebner phenomenon, indicating that the spots are more active in spreading. There are two main forms of vitiligo: segmental (unisegmental and ultisegmental) and non-segmental (acrofacial, focal, mucosal, generalized, universal, and mixed) (Rodrigues, Ezzedine, Hamzavi, Pandya, Harris, Group 2017). Segmental vitiligo does not follow normal rules and is distinct from all the other types listed above. It affects only one area of the body on only one side, without crossing the midline of the body on the front or back; but it is less common, affecting only about 5% of adults and 20% of children. It is rapidly progressive but stabilizes quickly, and is less responsive to treatment (Taïeb et al., 2007). In addition, it appears in non-segmental (generalized) or segmental on the skin, with varying patterns, and degrees of skin depigmentation (Armstrong, 2011; Ding et al., 2014). The challenges that vitiligo sufferers face are not limited to cutaneous symptoms and can also be associated to a range of other complications.

Vitiligo is problematic due to its psychological impacts, which are experienced by many patients around the world (Grimes & Miller, 2018). The levels of hopelessness, anxiety, depression, and general health of vitiligo patients have also been compared to normal controls in several studies (Hamidizadeh et al., 2020; Henning et al., 2020; Kussainova et al., 2020; Vernwal, 2017). Hamidizadeh et al. demonstrated that anxiety and hopelessness levels were significantly higher in vitiligo patients than those of healthy controls. Along with social or psychological distress, people with vitiligo may be at increased risk of sunburn, skin cancer, eye problems such as inflammation of the iris (iritis) and hearing loss (Hamidizadeh et al., 2020; Jakku et al., 2019).

Vitiligo is an autoimmune disease of the skin that results in a loss of melanocytes. Intrinsic defects in melanocytes may initiate disease through innate inflammation. Environmental factors also contribute, including exposure to phenolic compounds found in household products. Nonspecific induction of skin inflammation may induce local vitiligo lesions. The interferon-ʏ -CXCL10 pathway plays a central role in driving autoimmunity in vitiligo (Speeckaert et al., 2017). Genetics strongly influence the risk of developing disease (Seneschal et al., 2020). Genes encoding proteins mediating the immune response targeting melanocytes have been implicated in the etiology of this disease, together with proteins specific to these cells (Rodrigues, Ezzedine, Hamzavi, Pandya, Harris, Group 2017).

The etiology of vitiligo remains unclear, including the reasons for melanocyte death (Singh et al., 2016). It is underpinned by complex immune, genetic, environmental, and biochemical causes, however the exact molecular mechanisms of its development and progression are considered challenging to resolve (Liang et al., 2019; Sahoo et al., 2017; Singh et al., 2016). Although several vitiligo susceptibility loci have been reported using genome-wide association studies (GWAS), a study on monozygotic twins described the vitiligo concordance rate as 23% and suggested a remarkable environmental contribution to its pathogenesis (Singh, Lee, Vujkovic-Cvijin, Ucmak, Farahnik, Abrouk, Nakamura et al. 2016). Zheleva et al. revealed that oxidative stress is a triggering event in melanocytic destruction, which is probably involved in the enteropathogenesis of vitiligo disease. Oxidative stress biomarkers could be found in the skin and blood of vitiligo patients (Zheleva et al., 2018). Sahoo et al. reported that a human vitiligo cell line and PIG3V have unique lipid profiles, which are potentially associated with vitiligo activity in skin and blood. These profiles contain some biomarker candidates for determining treatment response and progressing the disease earlier and accurately (Sahoo, Lee, Boniface, Seneschal, Sahoo, Seki, Wang et al. 2017). Key proteins involved in vitiligo were surveyed for their molecular connectivity and topological parameters to identify causative factors in vitiligo. A comprehensive vitiligo map with 4845 protein nodes linked with 107,416 edges revealed a list of top-order proteins that play a key role in the disease pathology mechanism including SUMO2, ESR1, COPS5, MYC, SMAD3, and Cullin proteins (Malhotra et al., 2018). The need for a useful non-invasive tool for large-scale screening, urinary metabolomics was recently reported to detect disease-related metabolites using (Liu et al., 2020).

The role of other small molecules in vitiligo, such as amino acids, remains unanswered. In general, amino acids play an essential role in detoxification and immune responses through regulating the activation of T and B lymphocytes, natural killer cells, macrophages, and cellular redox state (Li, Yin, Li, Kim, Wu 2007). However, there is currently a dearth of comprehensive amino acid screening in vitiligo. Here, using metabolomics approach to assess global low molecular weight metabolites, we provide insights into the driving mechanisms of vitiligo and propose more potential biomarkers (Liang et al., 2019; Speeckaert et al., 2017). Our research focuses on establishing whether levels of important substrates, such as amino acids, are the most important primary metabolites altered in the plasma of vitiligo patients. Thus, these molecules may contribute to the vitiligo phenotype in melanocytes. In the present study we sought to evaluate a comprehensive profile of amino acids in the plasma of people with vitiligo compared to healthy people in order to find a fast-determinable potential biomarker, identify possible therapeutic target candidates, and to explore pathogenic pathways.

## Material and methods

### Patient samples

The study was undertaken according to the Helsinki Declaration and approved by the ethical review board of the Shahid Beheshti University of Medical Science with written and signed informed consent from the study subjects. Table 1 demonstrates patient and healthy individual data. Altogether 31 cases with vitiligo patients and 34 healthy individuals attended the dermatology clinic of Shohadaye Tajrish Hospital (Jun–Dec 2018). Vitiligo diagnosis was based on the characteristic loss of skin pigmentation and examination under a Wood's lamp. Plasma (with K2‐EDTA) was immediately separated from the peripheral venous blood by centrifugation at 1792 g at 4 °C for 20 min. The supernatant was isolated, snap-frozen, and stored at − 80 °C until analysis.

**Table 1:** Demographics of the study cohort

Information	HCs*	Vitiligo
Male	16	17
Female	18	14
Age, years**	35.8 ± 11.9	34.96 ± 11.59
Duration of the disease (year)	–	10.8 ± 9.5
Illness severity (body surface area involvement %)	–	32.41 ± 22.18
Active disease (having new lesions during last 6 months)	–	23
Positive family history	–	24

*HCs Healthy controls, ** = Means ± SD

### Amino acid analysis

To prepare the samples they were transferred from the − 80 °C freezer and placed on ice to be melted. To 50 µL of sample, 20 µL norleucine (500 μM) and 200 µL of methanol (kept at − 20 °C) were added and all are mixed for five seconds. To completely deproteinate, samples were kept at − 20 °C for 2 h, then centrifuged at 19,000 g for 12 min at 4 °C. The entire supernatant was transferred to a Heidolph rotary evaporator and dried in a vacuum. These samples could be stored at 4 °C for four weeks. For HPLC analysis, previously dried samples were dissolved in 100 µl of water (containing 0.01% formic acid). According to a previously described derivatization strategy, 10 µL *o*-phthaldialdehyde (OPA,) (for derivatization of primary amino acids) and 10 µL fluoronylmethyl chloroformate (FMOC-Cl) (for secondary amino acid derivatization) were added to 10 µl of each sample, and 20 µL of this was then injected into the HPLC system (Fekkes, 2012; Wu et al., 2016). For the HPLC–DAD method, a Knauer system (WellChrom, Germany) equipped with a K-1001 pump, a K-2800 diode array detector, an autosampler S3900 (Midas), a K-5004 analytical degasser, and a 2301 Rheodyneinjector with a 20 µL loop was used. HPLC separation was achieved using a Eurospher C18 column (4.6 mm × 250 mm, 5 µm), with a gradient elution program at a flow rate of 1.0 ml min^−1^. The mobile phase was composed of solution A (acetonitrile + 0.05% trifluoroacetic acid, v/v) and solution B (0.05% aqueous trifluoroacetic acid, v/v). Then, the following gradient was applied: 0–10 min, isocratic gradient 70% B; 10–30 min, linear gradient 70–40% B; 30–40 min, linear 40–20% B; 40–50 min, linear 20–0% B; 50–65 min, linear 0–70% B; 65–75 min, isocratic gradient 70% B. The chromatographic peaks of the sample solution were identified by spiking and comparing their retention times and UV spectra with those of reference standards. Quantitative analysis was carried out by integration of the peak using the standard external method. To detect primary amino acids, the fluorescence detector was set at 337 nm and 470 nm for adsorption and excitation, respectively. Also, detection of the first-type and the second-type amino acids was performed at 262 nm and 338 nm. The instrument stability of the analyses was checked during the study using quality control samples (QCs), a pool of all samples. This pool was prepared through mixing equal volumes of all samples and stored in 100 µl aliquots to avoid the repeated freeze–thaw cycles. A QC sample was injected in every six samples after being prepared under the same conditions. Measurement variability was assessed by calculating CVs based on QCs. All 22 amino acids showed acceptable repeatability with CVs < 10% in QCs. Also, as mentioned, the accuracy and precision of both derivatization and HPLC technique were performed using analyzing of five individual samples. The intra-day mean coefficient of variation (n = 3) and the inter-day mean coefficient of variation (n = 3) were within 2% and 7%, respectively.

### Statistical methods

We utilized R version 4.0.0 (https://www.r-project.org/) for our statistical study. The data was transformed into Log2 format before to the investigation. The Shapiro–Wilk test was used to check for normal distribution in the data. We used non-parametric approaches since the data were not normally distributed. To compare study groups, we employed the Mann Whitney U Test (Wilcoxon Rank Sum Test) with the BH (Benjamini Hochberg) correction (Mohieddin and Naser, 2019). We also used principal component analysis to gauge sample homogeneity (PCA). The importance of random forest features (mean Gini reduction) for the response variable was calculated to determine the difference between two groups. The confusion matrix was then calculated using the results of the random forest classification. We employed logistic regression and sensitivity analysis via the receiver operating characteristic (ROC) curve to examine the significantly different amino acids that we picked as biomarkers for the cause likelihood or severity of the disease. The Mann Whitney U test with BH correction was also used to compare the two groups of patients and healthy people by evaluating the link between disease severity, disease progression, and family history, with the amino acid profile. Furthermore, to explore the association of amino acids with disease progression, we divided vitiligo patients into two groups based on the percentages of skin involvement area. The 25% cut-off was chosen after evaluating a series of cut-offs using differential analysis of amino acid profiles. Therefore, the patients showing symptoms on more and less than 25% of their body skin surface area were categorized as progressive and benign vitiligo patients, respectively.

## Results

### Targeted metabolomic analysis of plasma reveals vitiligo‐specific biomarker profiles

**Fig. 1:**
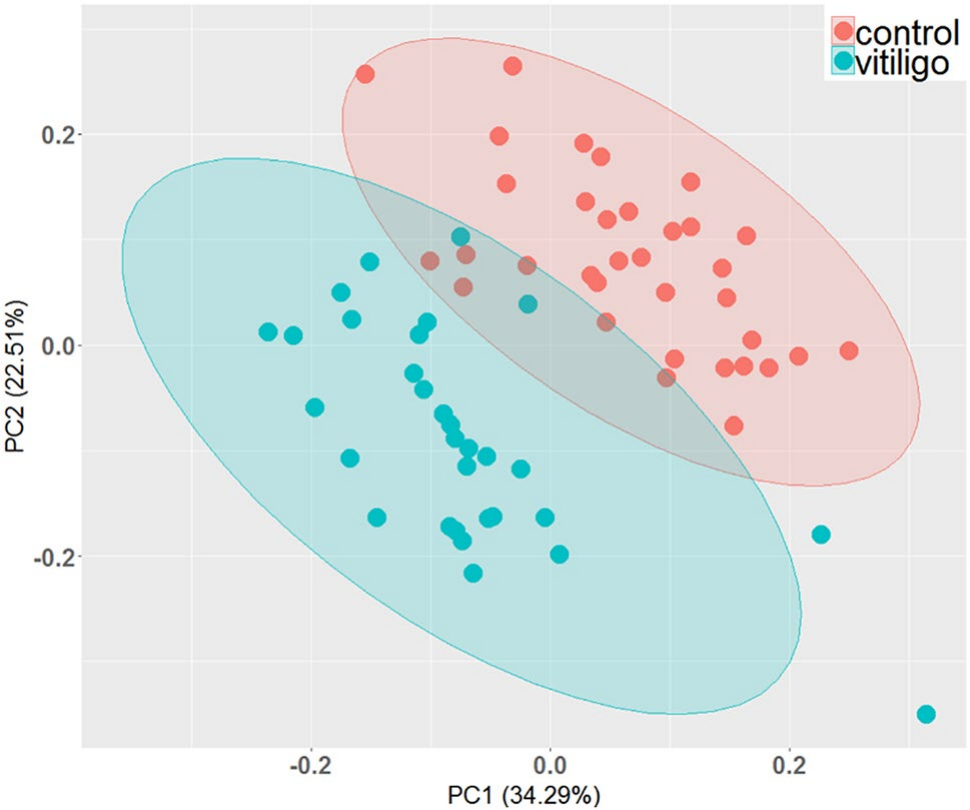
PCA analysis of data obtained by HPLC-FLD. All 22 amino acid concentrations were used to evaluate the sample homogeneity as a quality assessment of this study

We performed targeted quantitative analysis of 22 amino acids on the plasma of patients with vitiligo disorders. All samples from the patient and control groups (34 healthy cases and 31 vitiligo cases) were analyzed by principal component analysis (PCA). The samples were well-clustered in two separate groups (Fig. 1). Supplementary file 1 shows the absolute concentration of 22 amino acids in both groups.

**Fig. 2:**
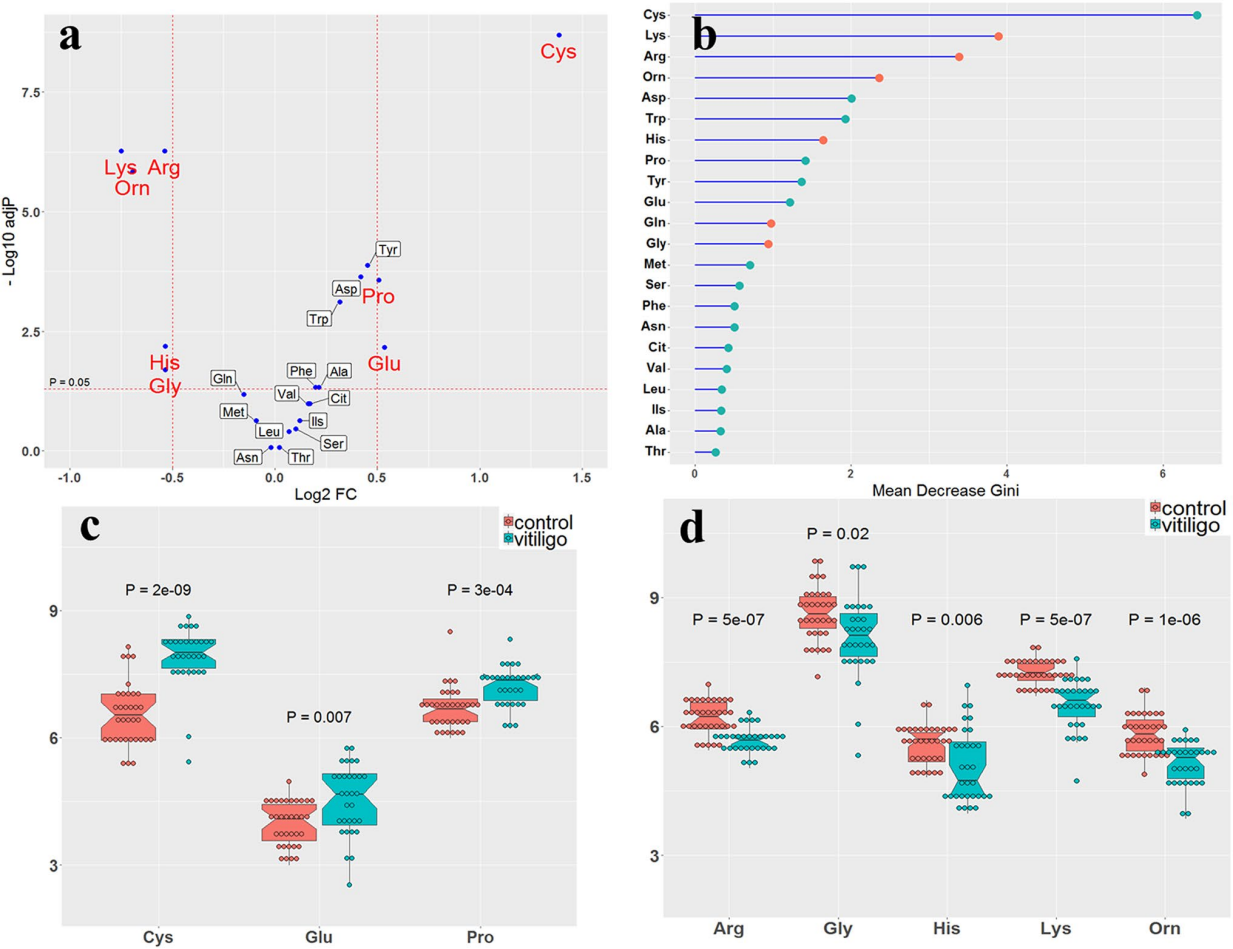
**a** Volcano plot of amino acid plasma concentration, **b** Gini error reduction diagram with tree number of 500, and notched box plot for increased **c** and decreased **d** amino acids. The red and blue dots indicate amino acid values ​​in healthy individuals (control) and vitiligo patients, respectively. The adjusted p-values ​​are represented in these plots

Next, the data were analyzed to find the difference in amino acid concentrations in both groups (Supplementary file 1 and 2). Figure 2a demonstrates a volcano graph in which horizontal and vertical axes correspond to log2 fold change of sample concentrations and − log10 adjusted p-values, respectively. The Wilcox rank test revealed a more than twofold elevation in Cysteine, Proline, and Glutamic acid levels with a p-value less than 0.05. In addition, lysine, arginine, ornithine, histidine, and glycine decreased by half or less in vitiligo patients (Supplementary file 2). Figure 2b shows the Gini error reduction diagram obtained from the random forest algorithm with a tree number of 500. In this plot, a higher mean decrease in Gini indicates higher amino acid importance. Amino acids are sorted and displayed in this plot based on the Gini index. Cysteine showed the highest Gini index with a high amount in vitiligo samples. Lysine and arginine were in the second and third rank of this plot with a high amount in healthy individuals.

**Fig. 3:**
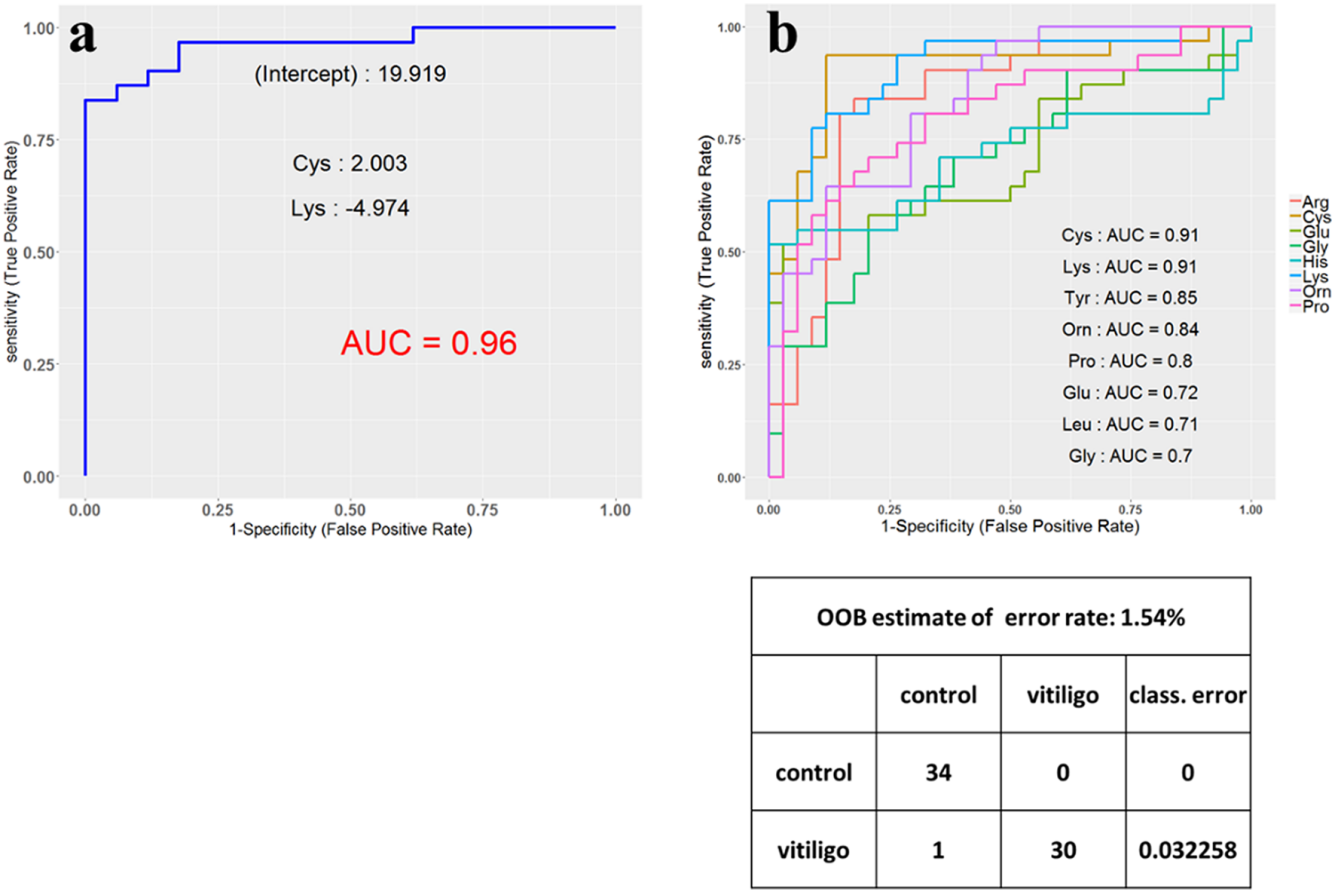
**a** ROC curve for Cysteine and Lysine with the highest variations, **b** ROC curve to show the sensitivity and specificity of the eight top amino acids, i.e. Cys, Lys, Tyr, Orn, Pro, Glu, Leu, and Gly independently with the AUC (Area under curve) values **c** confusion matrix based on random forest model

The performance of amino acids was then tested as disease biomarkers in a pooled set of vitiligo patients, to determine which amino acids would demonstrate the best sensitivity and specificity for vitiligo to be distinguished from healthy controls. Based on receiver operating characteristic (ROC) curve analysis, the top two significant amino acids with the highest area under the curve (AUC) were cysteine and lysine with 0.91 AUC independently and 0.96 AUC together, called “multi‐biomarker” for vitiligo (Fig. 3a & b). A positive/negative coefficient means the role of the amino acids in increasing or decreasing the risk of vitiligo. Next, based on the random forest method, a confusion matrix was developed in which two groups used in the present study were classified with a low amount of classification error (0.032) (Fig. 3c).

### Glutamic acid as biomarker for Vitiligo progressing

**Fig. 4:**
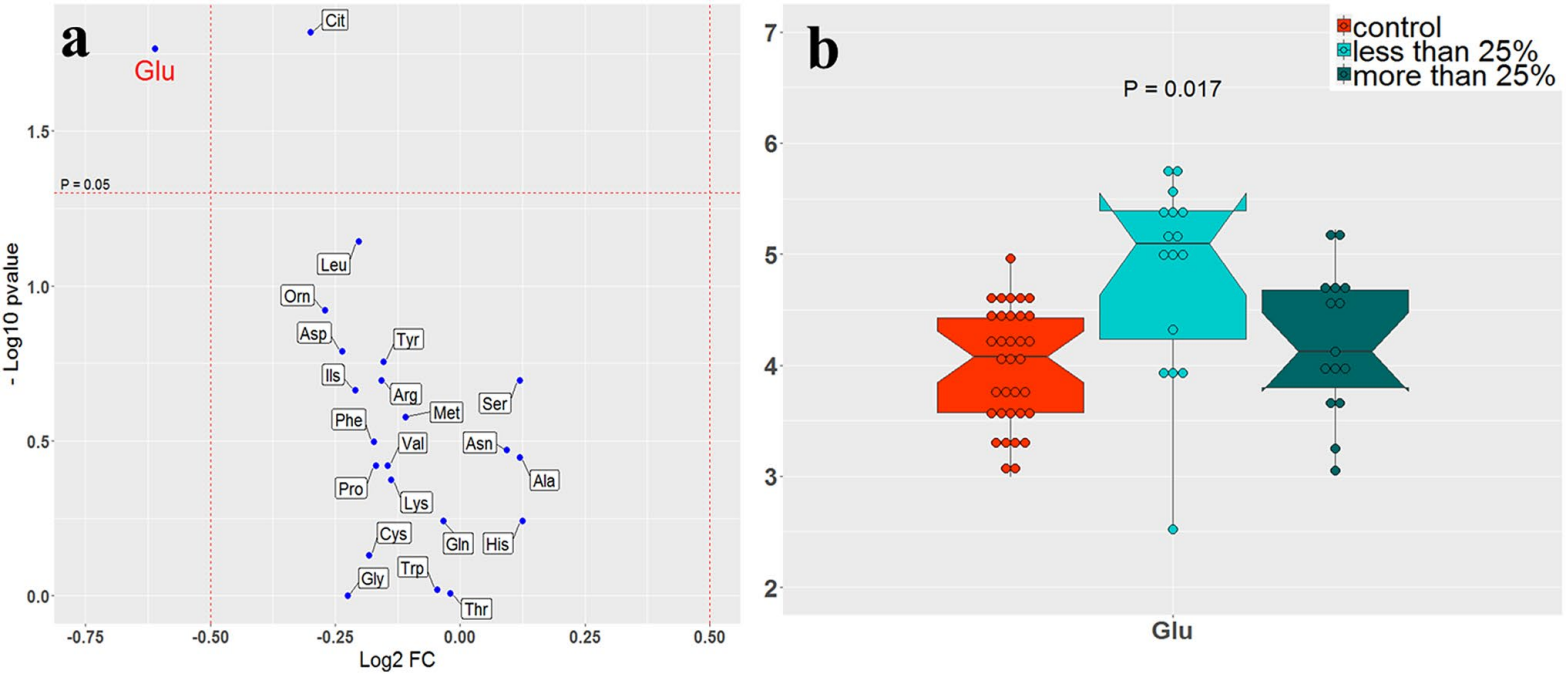
Amino acid amount analysis of patients with more and less than 25% of skin involvement area. **a** Volcano diagram shows the amount fold changes of all amino acids with adjusted p-values. **b** The notched box plots indicate the amount of glutamic acid in patients compared to healthy individuals

The role of amino acids in developing vitiligo was evaluated based on the size of skin involvement area. As a result, the proportion of vitiligo-affected skin compared to the total body area was utilized to assess any differences in amino acid amounts across multiple cut-offs. Glutamic acid had a lower amount by using 25% as a cut-off after comparing the patients with more and less than 25% involvement. It seems that glutamic acid becomes involved in developing vitiligo while it cannot contribute to vitiligo progress at a severe stage. The amount of glutamic acid decreased significantly for the patients with more than 25% involvement, similar to healthy individuals (Figs. 4a &b). It is worth noting that no other remarkable changes occurred in the amount of amino acids by considering a serial of cut-offs for skin involvement percentages.

Glutamate is easily the most abundant transmitter in the nervous system and is reported to be the neurotransmitter at 40% of all synapses in the brain (Binder et al., 2009). Therefore, if neuron fails to respond to glutamate, it is either not a neuron or it is dead. Glutamic acid decarboxylase (GAD) is an autoantigen in vitiligo and correlates with glutamic acid. Antibodies to GAD are frequently found in patients suffering autoimmune symptoms (Klemetti et al., 2000). This supports the critical role that glutamic acid may play in autoimmune diseases such as vitiligo.

### Pathway analysis: Amino acid metabolism remodeled in melanin production

**Fig. 5:**
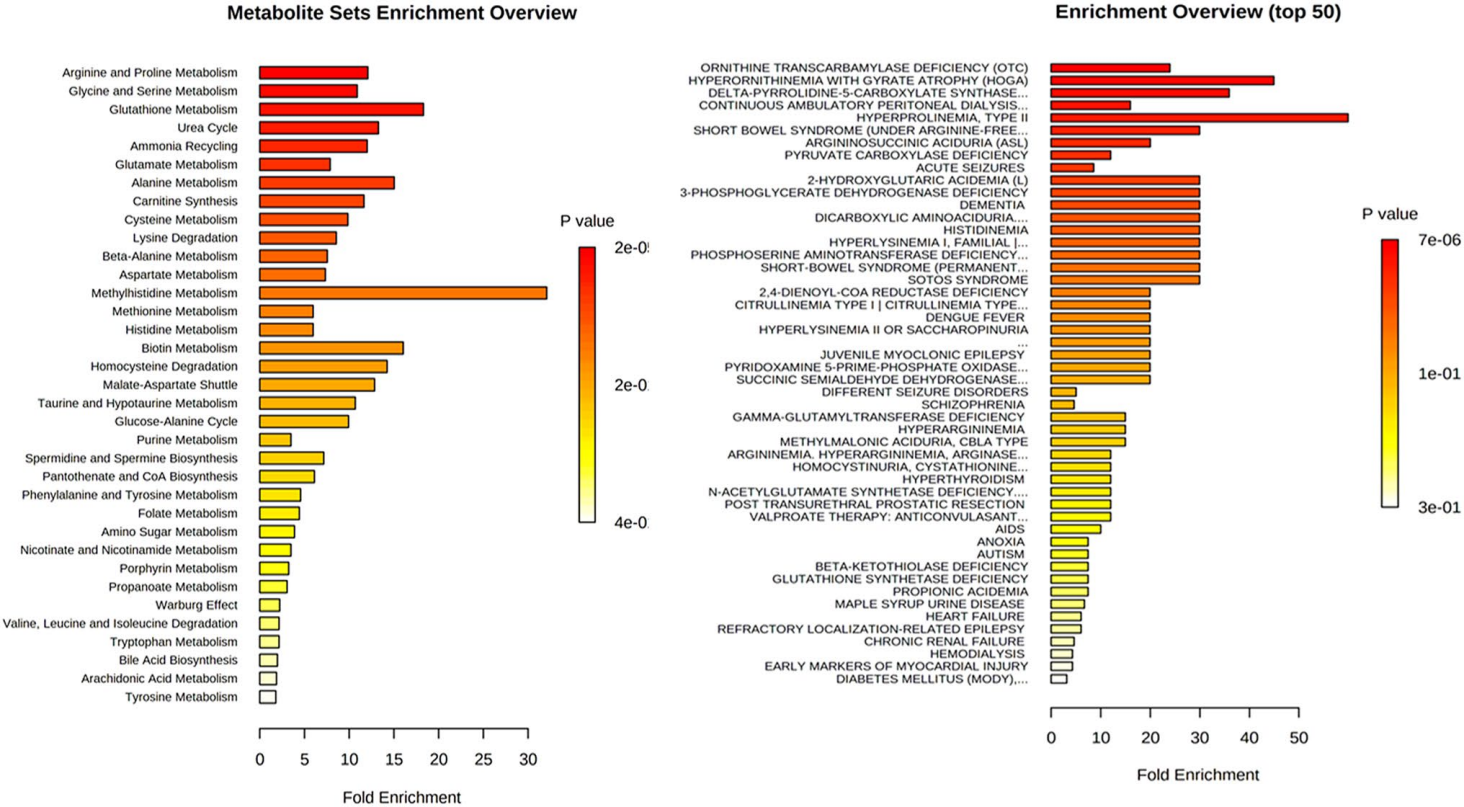
Pathway-associated and disease-associated metabolite analyses. The colored bars are based on P values, and the bar length corresponds to the fold enrichment. The corrected P values are provided

Pathway analysis of plasma amino acids showed several significantly changed pathways common to all vitiligo patients, but not to healthy individuals (Fig. 5). Pathway-associated metabolite and disease-associated metabolite analyses were performed to demonstrate the significantly altered metabolic pathways in Vitiligo cases. Based on the findings, 35 pathways were significantly different between Vitiligo and healthy samples, of which some pathways showed high fold enrichment values. Metabolites and metabolic terms changed in Vitiligo cases with lower p-values and higher fold enrichment values, including: arginine and proline metabolism, glycine and serine metabolism, glutathione metabolism, urea cycle, ammonia recycling, glutamate metabolism, alanine metabolism, carnitine synthesis, cysteine metabolism, lysine degradation, beta-alanine metabolism, aspartate metabolism, and methyl histidine metabolism. After disease-based enrichment analysis, disease-associated metabolite terms were observed for vitiligo patients compared to healthy controls. Ornithine transcarbamylase deficiency (OTC), Hyperornithinemia with gyrate atrophy (HOGA), Delta-pyrrolidone-5-carboxylate synthase, hyperprolinemia-type II, short bowel syndrome (under arginine -free), 2-hydroxyglutaric acidemia, 3-phosphoglycerate dehydrogenase deficiency dementia, dicarboxylic aminoaciduria, histidinemia, hyperlysinemia I-Family I, phosphoserine aminotransferase deficiency, short-bowel syndrome, and SOTOS syndrome are considered to be significant disease-associated metabolic diseases with high amounts of fold enrichment values.

## Discussion

The present study focused on disease‐specific amino acid fingerprints, which were detectable in the plasma of patients suffering from vitiligo, an asymptomatic disorder resulting from losing melanin. The disease group showed plasma amino acid fingerprints which were separately clustered from healthy controls, indicating the potential of amino acid fingerprints as multi‐biomarkers for diagnosis, disease progression follow-up, and possible treatment effect. Patients with skin involvement area more than 25% displayed similar amino acid changes reflected in plasma by providing more details about the role of amino acids in progressing the disease. Furthermore, the targeted metabolomics approach identified known therapy targets, already in clinical use, and identified new potential targets for treating vitiligo. Thus, targeted metabolomic analysis may be not only valuable for mechanistic studies, but also for metabolic targets in treatment trials. Amino acids play important roles in both mental and physical functions as regulators of metabolism, nutrients, neurotransmitters, and neuromodulators. There are many reports profiling amino acids to discovering biomarkers (Simińska & Koba, 2016). In previous studies of the metabolite and protein profiles of patients with vitiligo, the expression of several metabolites and proteins involved in the immune system were checked. Among these metabolites and proteins, lysophosphatidylcholine, platelet-activating factor, sn-glycerol-3-phosphocholine, succinic acid, CXCL4, and CXCL7 were significantly elevated in the plasma of patients with vitiligo. Interestingly, aspartate was downregulated and glutamic acid was upregulated (Liang et al., 2019). To the best of our knowledge, few studies have investigated the role of some amino acids and the derivative molecules associated with the melanin production in vitiligo such as phenylalanine, tyrosine and glucosamine, trimethylamine, cysteine, homocysteine, and thiol (Roy, 2007; Sahoo, Lee, Boniface, Seneschal, Sahoo, Seki, Wang et al. 2017). Based on these observations, there are both conventional and unconventional potential therapies for vitiligo, including: (i) amino acids such as L-phenylalanine; (ii) antioxidant agents such as alpha lipoic acid, glutathione (GSH), fluorouracil, levamisole, and melagenine; and (iii) metals such as zinc, minoxidil (Gianfaldoni et al., 2018).

However, we first provided the complete profile of free amino acids in plasma to evaluate the changes in the metabolic pathways of vitiligo. We found that arginine and proline metabolism are the most significant pathway enriched based on the altered amino acid profile in vitiligo. In most cell types, arginine, as a precursor of citrulline, regulates the activity of the immune system by producing nitric oxide (Rath et al., 2014). It is increasingly clear that proline metabolism plays an important role in metabolic reprogramming. Although first focused on proline catabolism, recent studies from a number of laboratories have emphasized the regulatory effects of proline synthesis and proline cycling. On the other hand, pyrroline-5-carboxylate dehydrogenase (P5CDH) is localized to the mitochondria and is critical in the anaplerotic role of proline released from proteins, for example, collagen (Phang, 2019). On the other hand, ornithine is catabolized by proline oxidase in different organs to produce hydrogen peroxide and pyrroline-5-carboxylate (P5C). By converting P5C into proline, a reduction occurs in the ratio of NADP + to P5C reductase-dependent NADPH (Abumrad & Barbul, 2004; Bansal & Ochoa, 2003; Flynn et al., 2002; Rath et al., 2014; Wu et al., 2005, 2011). Additionally, the proline-P5C cycle regulates the cellular redox state. These products have well-known functions, such as mitochondrial integrity, ion channel activity, cell death, antioxidation, and anti-tumor activity. Additionally, arginine and ornithine decreased and proline increased at the same time in patients. Thus, the findings indicated impaired arginine and proline metabolism, urea cycle, or nitrogen imbalance due to mitochondrial deficiencies in vitiligo. In other words, it is a consequence of a disruption in response to oxidative stress and cell damage. The amount of glycine in vitiligo was also lower than that of healthy controls. Glycine itself is considered as a potent antioxidant scavenging free radical, which is essential for the antioxidative defense of leukocytes. Furthermore, it plays a major role as an anti-inflammatory, immunomodulatory, and cytoprotective agent. Therefore, glycine reduction in patients strengthens the previously proposed association of developing vitiligo with the oxidative stress response. On the other hand, lysine, which decreases in vitiligo patients, has multiple catabolic pathways, such as carnitine biosynthesis. Carnitine and its esters help reduce oxidative stress (Pekala et al., 2011). Therefore, the reduction of this amino acid highlights the role of oxidative stress in vitiligo.

Additionally, glutamic acid increased in the patient group compared to the healthy control. It means that we can expect the increased activity of the immune system and the cellular redox state. Glutamic acid paves the way for transporting the reducing agents across the mitochondrial membrane and regulating glycolysis and cellular redox state through the malate/aspartate shuttle (Mori, 2007). In the present study, a high amount of cysteine was observed in vitiligo patients, which is the downstream product of homocysteine. Some studies indicated a high amount of homocysteine and thiols in vitiligo patients, and predicted the cysteine increment in this disease accordingly (Agarwal et al., 2015; Akoglu et al., 2018; Hamza et al., 2015; Sabry et al., 2014; Shaker & El-Tahlawi, 2008; Singh et al., 2011, 2016).

Tyrosine metabolism was also enriched in vitiligo patients. Tyrosine is converted into dopaquinone, as a highly intermediary metabolite, which is essential for regulating melanogenesis. Dopaquinone, in a rapid reaction with cysteine, becomes involved in pheomelanin production, which is considered a common melanin pigment found in hair and skin color (Ito et al., 2000). Likewise, we detected a high level of cysteine in the patients, indicating that the production of pheomelanin pigment is impaired by other factors, such as increasing thiol levels. Based on the findings, cysteine and the ratio of cysteine to ornithine increased along with a decrease in the ratio of glycine, arginine, ornithine, and lysine to cysteine in the patient group (Fig. 2 & Supplementary file 2). Thus, impaired cysteine metabolism disrupts the production of pigment, increases the activity of the immune system, and might counteract the effects of oxidative stress due to a deficiency in the production of antioxidant compounds.

Moreover, histidine was also significantly lower in the patient group. This amino acid plays a vital role in the skin vulnerability to the UV as an upstream molecule to make urocanic acid. In other words, histidine controls the activity of immune system against the UV radiation from the sun (Fabo & Noonan, 1983; Fabo et al., 1997; Gibbs et al., 2008); Hence, the reduced amount of histidine in vitiligo indicates a disturbance of the skin immune system, emphasizing the necessity of vitiligo patients to protect themselves against UV.

In the present study, glutamic acid was considered as a potential biomarker for progressing the disease. In addition, it can help determine 25% involvement of skin area as a candidate cut-off to divide patients into early and late stages of vitiligo. Furthermore, glutamic acid demonstrated a pulse propagation behavior by increasing the spot extent across the body surface. Due to the role of glutamate in regulating the cell cycle (Coloff et al., 2016; Murakami et al., 2012; Newsholme et al., 2003), its altering level in vitiligo can indicate implicated cell metabolism and increased cell death. On the other hand, some studies previously reported the influence of glutamate imbalance through glutamatergic neurotransmission with anxiety and stress (Amiel & Mathew, 2007; Bergink et al., 2004; Cortese & Phan, 2005). Our findings indicated that glutamate also engaged in this prevalent psychiatric disorder in patients, which has been described in vitiligo frequently (Hamidizadeh et al., 2020; Henning, 2020; Kussainova et al., 2020; Vernwal, 2017).

Interestingly, as shown in Table 1, 65% of the studied population has a positive family history. Vitiligo is a complex disorder (also termed polygenic and multifactorial), reflecting simultaneous contributions of multiple genetic risk factors and environmental triggers. Genetics influence the risk of developing disease (Seneschal et al., 2020). Early clinical case series reported a frequency of vitiligo in probands’ relatives of 11–38% (Spritz & Andersen, 2017). Also, there are some reports suggesting a polygenic, multifactorial mode of inheritance, and estimated vitiligo heritability at 46–72% (Hafez & Sharaf, 1983). Altogether the 65% of the studied population affected by vitiligo could be a consequence of both factors, including genetics and lifestyle.

## Conclusion

Based on the results of the present and previous studies on vitiligo, melanin production decreased by increasing the amount of cysteine and disrupted oxidative stress based on the glutamic acid and proline enhancement. Moreover, the reduction of arginine, glycine, lysine, histidine, and ornithine can damage melanocytes. They resulted in vitiliginous lesions on the skin surface of patients. This finding demonstrated the importance of these pathways either causing, or as a consequence of, vitiligo. Thus, examining the proposed biomarkers can contribute to the early diagnosis of at-risk patients. Additionally, considering the changes in glutamic acid levels as biomarkers can help determine the prognosis of the disease. In-depth understanding of the role of these biomarkers in vitiligo can provide the scientific basis for developing innovative therapeutic approaches for this disorder.

In the present study, separate patients were clustered based on their age‐ and gender‐matched controls in this targeted metabolomic analysis. The results highlight the potential of this approach for mechanistic studies and as biomarkers for disease progression follow-up. Despite the fact that our screening on amino acid targets corroborated previously examined metabolite targets for vitiligo disease and proposed new ones, these differential profiles would be best investigated at the localized tissue-level, which is the current study's limitation.

## Supplementary Information

Below is the link to the electronic supplementary material.

Supplementary file1 (XLSX 30 KB)

Supplementary file1 (XLSX 30 KB)

Supplementary file1 (XLSX 30 KB)

## Data Availability

Raw metabolite profiles and corresponding metadata are available from the MetaboLights repository (Haug et al. 2020) under the accession number MTBLS3462 (www.ebi.ac.uk/metabolights/MTBLS3462).
