# Supplementary figures and images for: Metabolomic signature of amino acids in plasma of patients with non-segmental Vitiligo
Source: Metabolomics. 2021 Sep 25;17(10):92. doi: 10.1007/s11306-021-01843-x (PMC8464575; doi:10.1007/s11306-021-01843-x)

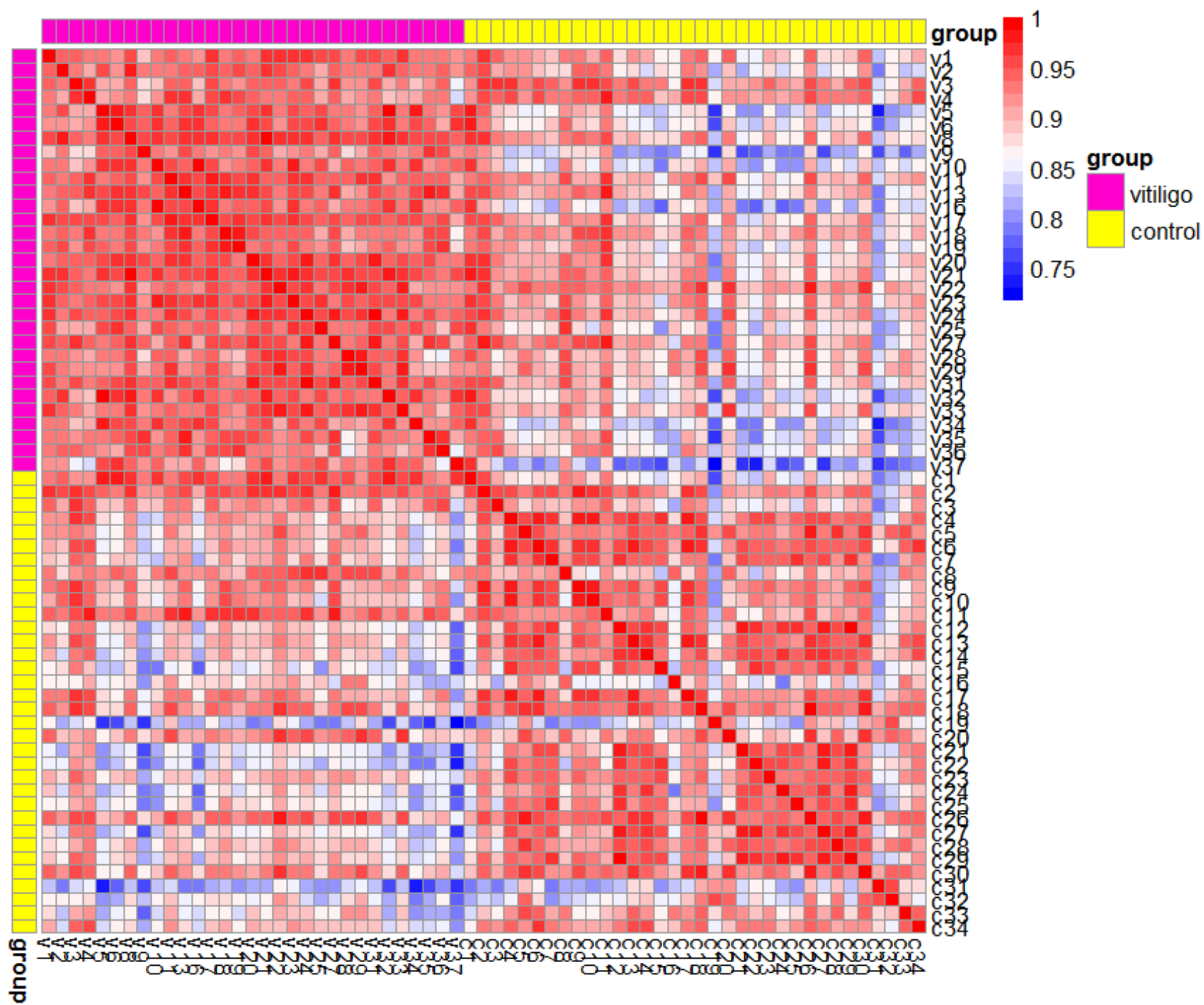

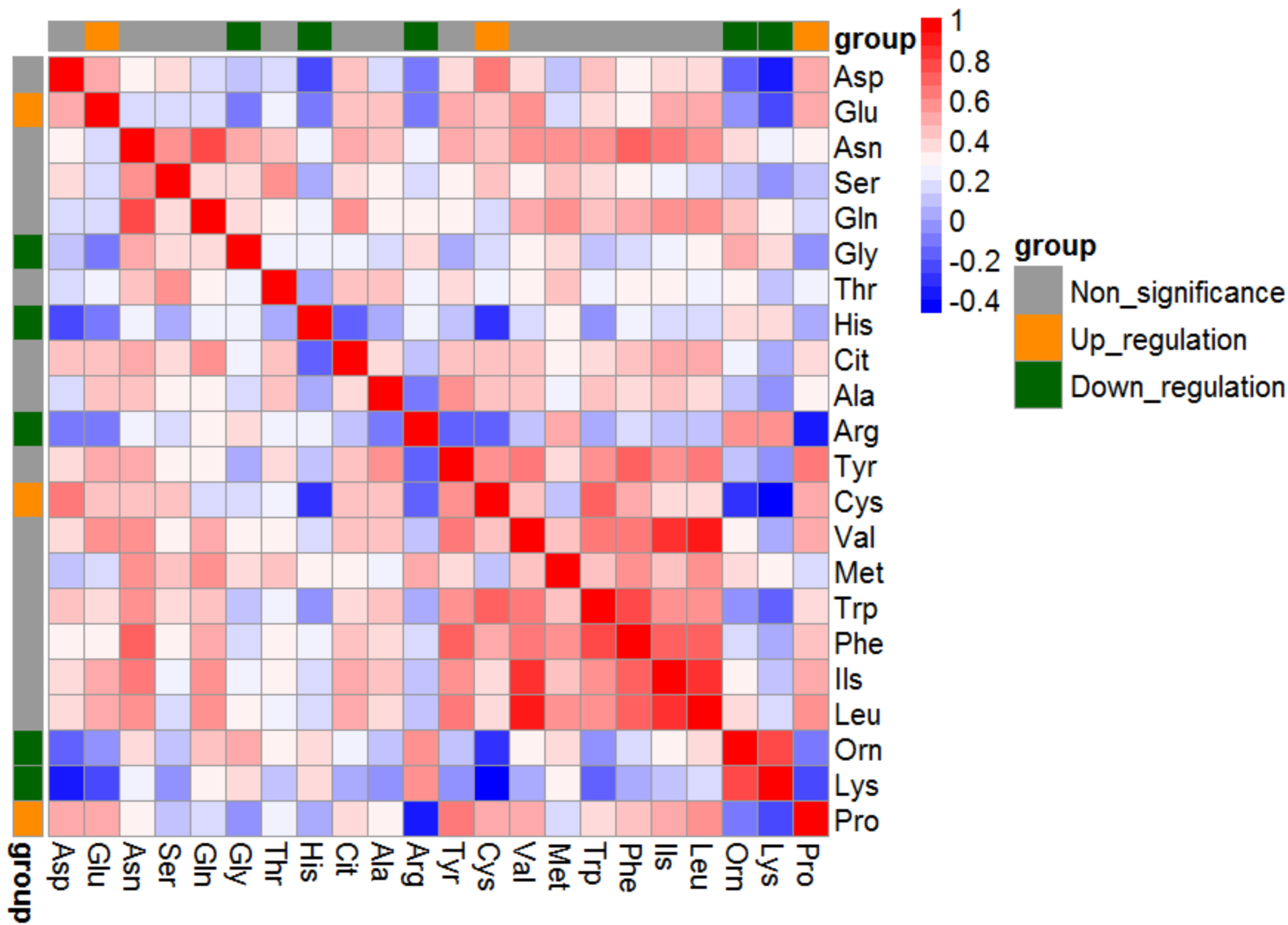

Supplement: Supplementary file 2 — Supplementary file1 (XLSX 30 KB) [file 11306_2021_1843_MOESM2_ESM.pdf]
